# Performance management: a qualitative study of relational boundaries in personal assistance

**DOI:** 10.1111/1467-9566.12996

**Published:** 2019-11-27

**Authors:** Tom Porter, Tom Shakespeare, Andrea Stöckl

**Affiliations:** ^1^ School of Health Sciences University of East Anglia Norwich UK; ^2^ London School of Hygiene and Tropical Medicine London UK; ^3^ Norwich Medical School University of East Anglia Norwich UK

**Keywords:** disability, care, direct payments, independent living, personal assistance

## Abstract

Personal assistance (PA) is a model of support where disabled people take control of recruiting, training and managing the people that support them. Personal assistance differs from other forms of care, such as domiciliary or informal care, because the disabled person is in control of how, when and by whom they are supported. With the advent of personal health budgets, PA is no longer limited to social care but is also central to future NHS services and funding arrangements. The aims of this study were to gain a deeper understanding of PA relationships, and to explore how both parties manage interpersonal challenges. We report on data from 58 qualitative interviews with disabled employers and personal assistants. Applying concepts from Goffman's (1959) scheme of impression management, we present an analysis of the relational dynamics that occur when two people cooperate in shared endeavours. Goffman's concepts of team members and non‐persons, in addition to the themes of regions and information control, aid a more fundamental understanding of the relational dynamics that occur between disabled employers and their PAs.

## Background

Personal assistance is a model of support where disabled people take control of recruiting, training and managing the people that support them. Personal assistance differs from other forms of care, such as domiciliary or informal care, because the disabled person is in control of how, when and by whom they are supported. In this respect, PA is opposed to traditional forms of care, which disabled scholars have identified as a form of oppression and an expression of prejudice (Morris 1997). Personal assistance, in contrast, is a principal tool for overcoming disabling barriers imposed by society (Mladenov 2012), and for empowering disabled people to make choices, enact autonomy and to take risks in ways that many non‐disabled people would take for granted (Marfisi 2002).

With the advent of personal health budgets, PA is not limited to social care but is central to NHS services and funding arrangements. Estimates suggest that in 2017, 70,000 people directly employed staff using local authority funding (Skills for Care 2017), up from 65,000 in 2016 (Skills for Care 2016). In the first 9 months of 2017/2018, 23,000 people received an NHS personal health budget (Department of Health and Social Care 2018a), up from 15,800 in 2016/2017 (NHS England 2017a). The latest NHS mandate sets the target of 50,000–100,000 people accessing personal health budgets by 2020 (Department of Health and Social Care 2018b).

One striking feature of PA in the UK is the relative lack of regulation governing its organisation. Whether funded privately, or through NHS and local authority direct payments, disabled people are free to employ workers and to organise their support with few restrictions (Priestley *et al*. 2006, Riddell *et al*. 2005). Other than fundamental duties of adult protection and situations involving regulated activities (NHS England 2017b), the personal and professional relationships that play out do so without oversight from government, professional or third sector agencies.

Boundaries act as the mould into which we pour our behaviours, tacitly guiding our feelings, gestures and actions in ways deemed appropriate to particular relationships. Yet unlike physical boundaries, relationship boundaries involve issues of power, influence and control (Austin *et al*. 2006). Boundaries therefore have two sides: they help us to understand what feels right, but their transgression may result in criticism or censure, and prompt feelings of shame, anger, even disgust. To date, limited research has addressed the types of relationships that develop within PA, or how disabled people and PAs understand and experience relational boundaries. In their study of PA relationships involving people with learning disability, Williams *et al*. (2009b: 621) report a ‘shifting tension between professional and personal identities’, something akin to what Woodin (2006: 12) terms ‘paid friends’. European studies apply similar labels in an attempt to capture the seemingly inherent tensions between professional and personal identities. In Norway, Christensen (2012) reports on ‘professional friendships’ marked by clear expectations upon both parties as set out in within employment contracts. Guldvik (2003) identifies two ideal types of PA – ‘Huma’ and ‘Pragma’ – each of whom prefer different types of relationship. Huma PAs seek affective attachment whilst Pragma PAs focus upon instrumental outcomes; one relationship is marked by intimacy, the other by professional distance. In Canada, Kelly (2010) has compared the influence of different underpinning philosophies; she finds that L'Arche communities promote PA relationships involving mutuality and shared lives, while disabled employers adapt the philosophy of Independent Living to reflect the reality of reciprocal PA relationships.

PA relationships develop over time (Glendinning *et al*. 2000), often becoming more sociable to the point that they resemble friendships (Woodin 2006). Indeed, disabled employers often cite friendliness as a marker of good PA. A sensitivity towards other people's emotions and the desire to communicate openly help both parties to work together (Ahlström and Wadensten 2011), whilst some disabled employers say that they actively seek PAs who demonstrate compassion or the potential for friendship (Matsuda *et al*. 2005). Less formal relationships are also likely to result in supportive arrangements that are more personalised and which feel less institutionalised (Williams *et al*. 2009a, 2009b). In PA relationships like this, flexible roles and expectations may lead to more reciprocal relationships, which deliver practical and emotional benefits (Leece 2006, Leece and Peace 2010).

But there are also risks accompanying less formal PA relationships. Research in Sweden has shown that informal PA relationships may lead PAs to feel responsible for their employer's support arrangements (Ahlström and Wadensten 2012). Alternatively, PAs may struggle without clear directions as to how they should feel and act whilst supporting someone they care about. Indeed, PAs have reported moral and emotional dilemmas as they attempt to define the personal and professional parameters of their relationships (Ahlström and Wadensten 2010). Another ideal image of PA promotes their ‘invisibility’ as virtuous; much like other supportive aids, such as wheelchairs, going unnoticed is a mark of quality. Yet this invisibility denies voice and recognition of workers who are often young, female and working in low‐wage and insecure roles. This dynamic risks a situation whereby the liberation of disabled people is advanced through the concomitant disenfranchisement of others (Neumann and Gundersen 2018).

Critical disability studies draw further attention to the interconnections between identity, embodiment and the politics of selfhood in PA relationships. Rejecting the notion of ontologically delimited bodies and beings, post‐structuralist studies challenge traditional readings of supportive relationships and the veracity of autonomy and independence as emancipatory goals (Fritsch 2010, Gibson 2006). Personal assistance, from this view, is inherently transgressive because it subverts putative norms of embodied and affective comportment.

## Theoretical lens

Goffman's (1959) analysis of impression management guides our interpretation of boundaries in PA. Our focus is not the performative aspects of PA per se, but the micropolitics that occur when two people engage in collective endeavours. The dramaturgical lens reveals interpersonal dynamics of cooperation and conflict within social dyads, whilst Goffman's concepts of ‘team members’ and ‘non‐persons’ shed light on how the different ways that PAs relate to their employers. Furthermore, Goffman's themes of information control and regions further elucidate the management of boundaries.

In *The Presentation of Self in Everyday Life* (1959), Goffman's central concern was impression management, or the manner in which individuals seek to maintain face to others. Conceived as performance, impression management involves performers and audience members, as well as a supporting cast of individuals each playing distinct roles in relation to the performance. Most prominent among this supporting cast are team members, whose role it is to foster and sustain performance through intimate cooperation with the primary performer. The form and function of cooperation varies, but relationships between team members are characterised by bonds of mutual dependence, and the fact that team members hold a personal stake in the success of the performance. In Goffman's terms, the role function of team members is firmly that of a performer, not an audience member.

Another of Goffman's analytic themes is regions, defined as ‘any place that is bounded by some degree by barriers to perception’ (Goffman 1959: 109). ‘Front regions’ are those accessible to both audience members and performers alike and are the places where impression management plays out. Because front regions fall under the gaze of audience members, performers must maintain comportment in ways consistent with the impression they seek to convey. Back regions, or ‘backstage’, are places accessed only by entrusted members of the performer's team. These regions; be they spaces, settings or occasions, are firmly off‐limits to audience members. In backstage places, performers no longer need to conduct themselves as though they were in the presence of audience members – they may ‘be themselves’ as impression management is dropped.

Information management is also key to Goffman's analysis and the extent to which actors hold information and secrets about one another will define their relationship. The control of information is crucial to the success or failure of any given performance, whilst disclosing (and being privy to) personal information builds trust and complicity between actors. Team members characteristically hold insider knowledge about their fellow performers and are their accomplices, ensuring the security of discrediting information.

The relationship between role functions, regions and information control is not always straightforward, however, and Goffman's scheme includes further roles that are neither performers nor audience. These Goffman terms ‘discrepant roles’ because they have access to information and regions in ways that diverge from the standard performer–audience dynamic.

One class of discrepant roles are ‘non‐persons’ – individuals that are present during interactions, but whom neither performers nor audience members recognise as full persons. Examples of non‐persons include domestic staff and service specialists (such as bar staff or hairdressers). Such roles have much in common with team members: they are present in front regions during performances; they have access to back regions and they are not people towards whom face must be maintained. Yet unlike team members, actors in these roles do not hold a stake in the success or failure of the performance. As Goffman states, they do not share ‘the risk, the guilt, and the satisfaction of presenting before an audience the show’ (Goffman 1959: 153). More importantly still, despite having access to back regions and being privy to the secrets of others, performers do not learn corresponding secrets about, nor access the backstage regions of, those in these roles. This is their discrepant quality.

Personal assistance, much like performance, involves the cooperation of two people in shared endeavours. Goffman's concepts of team members and non‐persons, in addition to the themes of regions and information control, aid a more fundamental understanding of the relational dynamics that occur between disabled employers and PAs. We introduce these concepts throughout our findings, before returning to a fuller exploration of this analytic scheme in the final discussion.

## Study methodology

### Sampling and recruitment

Disabled informants were sampled purposively on the basis that they had experience of employing PAs. Exclusion criteria included being under the age of 18 years and lacking mental capacity to provide informed consent. PA informants were recruited initially through disabled people's organisations (DPOs) and online forums, and subsequently using snowball sampling. Informants from England, Scotland and Wales participated in the study, although country of residence was not a sampling criterion. PA participants were recruited initially through DPOs and subsequently through snowball sampling.

Participants recruited through DPOs were contacted by representatives from the DPO, who introduced the study and provided information sheets and consent forms. Participants recruited through snowballing, and those who responding to online study adverts, initiated contact with the research team. After making contact with the research team, either by post, email or telephone, all participants had opportunity to ask questions about the study. The researcher ensured that each participant understood what involvement would entail. Informants gave informed consent prior to each interview and researchers reaffirmed this after the interview had finished.

The sample of employers included 19 women and 11 men. Disabled participants were asked to define their impairment, the details of which are included in Table 1. Included in this sample are three parents who employ and manage personal assistants on behalf of children under the age of 18. The sample of personal assistants included 22 women and 6 men (Table 2).

**Table 1:** Disabled employer information

ID	Sex	Interview type	Self‐defined impairment	Ethnicity
DP01	F	Face‐to‐face	Familial dysautonomia	White British
DP02	F	Face‐to‐face	Spinal cord injury	White British
DP03	F	Face‐to‐face	Multiple sclerosis	White British
DP04	M	Face‐to‐face	Multiple sclerosis	White British
DP05	M	Face‐to‐face	Cerebral palsy	British Asian
DP06	F	Face‐to‐face	Cerebral palsy	Black British
DP07	M	Face‐to‐face	Cerebral palsy	British Asian
DP08	F	Face‐to‐face	Muscular dystrophy	White British
DP09	M	Telephone	Musculoskeletal condition (non‐specified)	White Non‐British
DP10	F	Telephone	Physical impairment	White British
DP11	F	Face‐to‐face	Phocomelia	White British
DP12	M	Telephone	Multiple sclerosis	White British
DP13	F	Face‐to‐face	Physical impairment (non‐specified)	White British
DP14	M	Face‐to‐face	Spinal muscular atrophy	White British
DP15	F	Face‐to‐face	Myalgic encephalomyelitis	White British
DP16	M	Email	Physical impairment (non‐specified)	White British
DP17	F	Face‐to‐face	Spinal muscular atrophy	White British
DP18	F	Face‐to‐face	Friedreich's ataxia	White British
DP19	F	Face‐to‐face	Spinal cord injury	White British
DP20	F	Face‐to‐face	Multiple sclerosis	White British
DP21	M	Telephone	Physical impairment (non‐specified)	White British
DP22	F	Face‐to‐face	Multiple sclerosis	White British
DP23	M	Telephone	Physical impairment (non‐specified)	White British
DP24	F	Telephone	Multiple sclerosis	White British
DP25	F	Face‐to‐face	Myalgic encephalomyelitis	White British
DP26	M	Face‐to‐face	Muscular dystrophy	White Non‐British
DP27	F	Telephone	Mother to daughter with Down's syndrome	White British
DP28	F	Face‐to‐face	Mother to son with learning disability	White British
DP29	F	Telephone	Mother to son with Down's syndrome	White British
DP30	M	Telephone	Physical impairment (non‐specified)	White British

**Table 2:** Personal assistant information

ID	Sex	Interview type	Ethnicity
PA01	F	Face‐to‐face	White Non‐British
PA02	F	Face‐to‐face	White British
PA03	F	Face‐to‐face	White British
PA04	M	Face‐to‐face	White British
PA05	F	Face‐to‐face	British Asian
PA06	M	Face‐to‐face	White Non‐British
PA07	M	Telephone	White British
PA08	F	Face‐to‐face	White British
PA09	M	Telephone	White British
PA10	F	Telephone	Black Non‐British
PA11	F	Telephone	White British
PA12	F	Telephone	White British
PA13	F	Telephone	White British
PA14	F	Telephone	White British
PA15	F	Telephone	White British
PA16	M	Telephone	White British
PA17	F	Telephone	White British
PA18	F	Telephone	White British
PA19	F	Telephone	White British
PA20	M	Telephone	White British
PA21	F	Telephone	White British
PA22	F	Telephone	White British
PA23	F	Face‐to‐face	White Non‐British
PA24	F	Telephone	White British
PA25	F	Telephone	White British
PA26	F	Telephone	White British
PA27	F	Telephone	White British
PA28	F	Telephone	White British

A limitation of this study is that it did not recruit young people or disabled adults with intellectual disabilities. PA relationships involving children or disabled people with intellectual disability are likely to be distinctive, therefore these limitations are regrettable. However, there exists significant and high‐quality research into these kinds of relationships in the UK context (Williams *et al*. 2009a, 2009b).

### Data collection and analysis

Qualitative interviews were chosen because the study was concerned with the meaning of PA relationships and understanding how participants made sense of their experiences (Brinkmann and Kvale 2015). Data collection took place between 2015 and 2017. Three types of interview were offered: face‐to‐face, telephone and email. The majority of disabled informants took part in face‐to‐face interviews, all but one of which took place in informants’ own homes, with one conducted in a public space chosen by the participant. Most PAs took part in telephone interviews, largely because they were more geographically dispersed. Interviews lasted between 30 minutes and 3 hours.

Interviews followed a topic guide informed by literature and refined iteratively throughout data collection. Where interviews were conducted by email, informants were sent a document containing a topic guide, which they annotated and returned to the research team. All members of the research team conducted interviews, which were transcribed verbatim. Data storage, administration and analysis were conducted using QSR Nvivo 11 (QSR international, https://www.qsrinternational.com/nvivo/contact-us/contact-form).

Constructivist Grounded Theory (Charmaz 2014) provided the framework for analysis. The first stage of coding was initial coding followed by increasingly directed and conceptually driven focused coding. Following Charmaz, focused coding involved identifying and expanding the most theoretically significant and frequently occurring codes delivered through initial coding. A final stage of theoretical coding analysed categories of codes generated through focused coding. In practice, as recognised by Charmaz (2014), the distinction between each coding stage was flexible, and coding was an emergent process – as concepts emerged, initial coding was revisited and re‐coded in light of subsequent theoretical coding. Coding accuracy and interpretation were cross validated by all members of the research team.

The host institution's Faculty of Medicine and Health Sciences research ethics committee provided ethical approval for the study. The study was funded by the Economic and Social Research Council.

## Findings

Three main themes emerged: informality as inevitable and advantageous; the risks of informality and PAs protecting the private realm. The second of these themes has four subthemes: practical problems, emotional entanglements, ethico‐legal considerations and giving too much. PAs relate to their employers in different ways based largely upon whether they assume the role of team member or non‐person. All informants made sense of boundaries by reference to regions and information control. Data from disabled participants and PAs are labelled DP and PA, respectively, with both groups numbered sequentially.

## Informality as inevitable and advantageous

Many disabled informants said that formal PA relationships were neither possible nor desirable, as PA work fostered informality and emotional attachment. Intimate tasks and the disclosure of personal information were two aspects of PA work commonly identified as precipitating informal or personal relationships. Informant DP03 said that it was ‘the nature of the job’ for PA to involve friendship, and in doing so, made reference to personal care and the trust needed to complete such intimate work:

They're doing quite personal things like showering you. They've got to be people you trust intimately and people that you know, very, very well. And because of that, you've got that relationship going.

Informant DP20 said that relying upon other people for support made the sharing of personal information unavoidable: ‘I literally can't do things, and you have to tell them things because that's what happens to your body’. Such disclosures meant that for DP20, the distinction between personal and professional realms was unrealistic: ‘people know things about me that they wouldn't know in any other circumstance’.

Spending prolonged periods of time in one another's company, and the fact that a PA's work place is their employer's home space were further drivers of informality. Informant DP07 said it was ‘natural’ for PAs to become more than just staff, saying:

you're always going to become some kind of friend … because you're working so closely … you're spending up to 72 hours in each other pockets.

PA informant PA16 said ‘if you are with somebody so much, it is very easy to drift into friendship’. Another PA informant, PA06, said that working in the home space made for more relaxed situations:

It's not as if you are relating to your colleague at work or your boss at work, you are part of their daily routine and you get to know their most personal needs … you are in the person's house and into their lives completely.

These excerpts show clearly that PAs routinely access the backstage regions of their employer's lives, whilst disabled employers are often required to disclose personal information to their workers. Both these factors shape the relationship, as social distance is reduced and informality grows.

Disabled informants also spoke about the instrumental benefits of less formal relationships, which they said were more relaxed, easier to manage, and less disruptive to the home space. Informant DP10 said that formal relationships were impractical and incompatible with her desired vision of the home: ‘I'm a human being and I can't act like I am an employer all the time’, adding ‘this is my house, I have to be able to relax in my own home’.

Instrumental benefits were also reported by Informant DP03, who said:

This is where the friendship comes in, because she's a friend and she wants to do something for me, she wants to help me, and being friends they want to help you. Not being told to do something because they're working for me, but because they're friends.

In Goffman's terms, the PA relationships outlined here resemble those of team members. PAs cooperate in the shared goal of their employer's independence, and hold an affective stake in the success of this endeavour. Moreover informant DP10's expressed desire to ‘relax in my own home’ indicates that she does not consider her PAs to be audience members, and thus need not maintain the face of an employer in their presence.

## The risks of informality

Despite the benefits of informality, nearly all informants spoke about problems they had encountered in less formal relationships. Disabled informants emphasised practical problems and emotional entanglements, whilst PA informants told of ethico‐legal dilemmas and the prospect of giving too much of themselves – both physically and emotionally – to their work.

### Practical problems

Many disabled informants recounted experiences of unsatisfactory support within friendly relationships. Informant DP10 said that she took care not to ‘fall into the trap of being a bit easy on people’, and cautioned that friendship allowed PA standards to slip: ‘when they feel they've got their feet under the table … they try to cut corners’. Similarly, informant DP11 reasoned ‘at the end of the day the PA is there to be a PA … you don't want it to be forever’. This informant was clear that she prioritised performance over friendship, saying ‘you want consistency, I don't want someone there forever because they run out of steam’.

Disabled informants also said that friendship made it harder to act assertively towards PAs. Informant DP21 said that giving close instructions felt ‘awkward’:

You have to ask them to do things, but if they don't want to do it, or if they've done something wrong, you have to say to them “no I don't want it done like that.”

Similarly, informant DP08 expressed unease at the prospect of disciplining her PAs, saying ‘I am not a massive fan of confrontation’. Such sentiment is understandable given that friendship involves respect and care for another; giving orders or delivering discipline requires the overt exercise of power, which can feel inappropriate in more convivial relationships. Some disabled informants in this study had previously employed friends or family members as PAs, but most felt this was unfeasible. Informant DP07 said ‘if a friend becomes your PA it makes it messy’, adding:

It's hard to dismiss someone because you have an emotional attachment as well as the professional side. Also, you've got the fear of losing a friend.

Informant DP30 said that employing friends ‘just didn't work’, adding ‘it's just not feasible … they take the piss’. As with other PA users, this informant explained that informality and conviviality could easily lead PAs to lower their standards, whilst also making it more difficult to enact authority. Consequently this informant stated a clear preference for employing strangers rather than friends, saying of strangers ‘it's a lot easier to assert professional control over them’.

Being able to relax and not having to maintain face in front of PAs was important for most disabled informants. However, for some, the performance of PAs in roles resembling friendship – team members – fell short of their expectations. This is likely the result of role dissonance, as PAs struggle with the expectation to maintain instrumental face, whilst their employer need not, or cannot, maintain the face of formal employer.

### Emotional entanglements

Both disabled and PA informants spoke of close PA relationships involving difficult emotional entanglements. Informant DP07, who fell in love with one of his PAs, provided the clearest example of this. Speaking of the relationship, he said ‘there weren't clear boundaries, which neither of us set … it got very confusing for both parties’. His romantic feelings were unreciprocated, and DP07 described the difficulties that followed: ‘It was quite traumatic … there were tears, letters and emails, and trips to the airport. It was quite messy’. Despite this painful experience, informant DP07 remained sanguine, saying ‘it made me realise how important it is to have boundaries and to understand the relationship between the PA and a PA user’.

A small number of disabled informants described onerous emotional work (Hochschild 1979), undertaken whilst supporting their PAs. Informant DP10 said that she had previously ‘fell into being a little bit too caring’, meaning that her PAs came to rely on her support: ‘I had people texting me all hours of the day and night, which was a bit ridiculous’.

Many PA informants also reported emotion labour (Hochschild 1979), particularly in relationships involving mutual affection. In one clear example, informant PA01 said: ‘you don't really finish your shift and finish your work’. This informant explained that she felt unable to separate her work and home life, saying ‘I go home and think “oh, she said this today … I wonder what she meant with that?”.

Other PAs said that emotional pain was more likely in close relationships. Informant PA13 spoke fondly about the ‘lovely relationship’ she shared with her employer, whom she described as ‘like a mum’. This informant explained that her employer's health had recently deteriorated, and when asked how she felt about this, she replied:

It feels like it is my mum having a bad turn … I will feel awful when she dies, or if she has to go into a home. We talk about this, she definitely doesn't want to go into a home, and I will feel really bad.

Another PA informant, PA22, described feeling ‘injured’ after being admonished by her employer. This informant recalled an argument between her employer and her employer's daughter, after which her employer criticised PA22 for passing judgement on the daughter's behaviour: ‘I explained how I felt about what had gone on … she turned to me and harshly said “she doesn't need you parenting her.” Describing how this made her feel, PA22 said ‘I felt really injured by that’. When asked why she had felt this way, PA22 pointed to the close relationship she shared with her employer: ‘[it's] because I care passionately about my client, because that matters to me, it so matters … therefore it is important to me’.

### Giving too much

Nearly all PAs expressed concerns about working beyond their paid hours, with many suggesting this was more likely in relationships that resemble team members. Informant PA08 said ‘you have to create a boundary between the friendship role and the professional role … being able to be clear about how much you can physically do because someone can just push you and push you’. This informant explained that being ‘pushed’ by an employer involved emotional as well as physical work: ‘often people [employers] are quite lonely, they don't interact much … you can become on the receiving end of a lot of emotional stuff, which I find can be very exhausting’.

Informant PA03 said ‘it's easy to get sucked in and over commit’, adding that this could be ‘at detriment to yourself’. This informant explained that she cared for her employer and felt responsible for her wellbeing, and described the work she undertook to ensure her employer's support:

you can become totally overwhelmed by the whole situation, if you are being called on too much and you've allowed yourself to be pulled into a situation where you are doing more and more hours … you can be overwhelmed.

Describing her work as ‘such an essential thing’, informant PA19 said that she often worked more shifts than she desired. This informant found it impossible to remain disinterested in her employer's wellbeing, meaning she felt unable to turn down her requests for help. Describing a recent working week she said ‘I was supposed to work one 12 hour shift, I ended up working four’. When asked whether she planned to continue working as a PA, this informant said ‘I don't see myself doing it’ and explained that she no longer wanted to feel responsible for her employer's support arrangements: ‘you get committed and there are always crises, crises always occur and you really want to help’.

In rare cases, PAs described intensely personal relationships in which their employer's wellbeing appeared to supersede their own. In one example, the bond between informant PA21 and the child she supported was such that she dedicated significant unpaid time and energy to his support. The child in question had recently been withdrawn from school, and PA21 described the extent of her involvement in his support:

I was teacher, PA, everything … officially I was teaching him 15 hours a week, 3 hours every morning, but then I was doing other activities, so I had him 6 days a week. A lot of hours.

This informant received pay for 24 hours work each week, but when asked how many hours she actually worked, she replied ‘at least double that’. Explaining why she committed so much of her own time, PA21 said:

I suppose I worked with him for so long, invested everything … it has gone further than just a PA job. I knew I could help him, so I had to. I couldn't watch him carrying on down the path, I knew where that would end up. I had to do something.

For informant PA21, this commitment was a natural and rewarding feature of a deeply committed personal relationship. But thinking dispassionately, this example also illustrates ethical dilemmas concerning the appropriateness of such close PA relationships, and raises the question of whether PAs receive adequate remuneration for such unacknowledged work.

Emotional work, emotional labour and ‘giving too much of oneself’ are more common in PA relationships where a bond of mutual affection develops and where PAs invest personally in lives of their employers. In such relationships, PAs hold a personal stake in the success or failure of their shared endeavour. These are characteristic features of team member roles, and suggest that the risks of emotional entanglement and giving too much are inherent to these relationships.

### Ethico‐legal considerations

Several PAs drew upon discourses of legal and professional ethics when making sense of complex relational dilemmas. For some, particularly those with a background in traditional care roles, the logic of safeguarding meant that emotional attachment and informality were deemed inappropriate. These PAs clearly understood the nature of their role differently from PAs assuming team member roles. Instead, for these PAs who resemble Goffman's non‐persons, social distance is maintained by professional ethics inherited from formal care roles.

When asked whether it was possible to separate tasks from emotions, informant PA07 said ‘yes, definitely. They have to be’. This PA felt that emotional detachment was vital for her to meet what she understood to be her legal duties: ‘I am bound by legislation that would require me to make a report to someone if I felt something wasn't right’. This informant, who had previously worked for a care agency said ‘I can't get emotionally involved in the tasks, because then my objectivity would be clouded’.

Similarly, informant PA17, who had also worked as domiciliary carer, said that she would not complete tasks without having received formal training: ‘there are certain things I won't do. I go to one lady and she needs a suppository for her medication, and I won't do that because I am not trained’. This standpoint had caused tensions among other PAs, as PA17 felt the conduct of others was unprofessional: ‘I think you can be seen as a bad person, but actually you are doing things by the book’. When asked why it was important to ‘do things by the book’, PA17 replied: ‘well, something could become a safeguarding issue, and if there was an enquiry … it's about covering your back at the end of the day’.

PA informant PA06 said that ‘finding the boundary between personal and the professional’ was the ‘biggest challenge that I found’. This informant spoke in depth about one PA relationship, in which he supported a young man who lived with his mother. PA06 felt that the young man in question was being constrained by his mother, who he felt was controlling and overprotective. He explained the dilemma he faced:

I felt he should somehow make himself heard with mother, that he was capable of things … I really wanted to help him in this fight, I felt that he was feeling the same things that I felt but he couldn't actually externalise them.

Despite this, PA06 felt that his intervention would be inappropriate: ‘I realised that it wasn't my place to be doing this, it wasn't part of my job’. For PA06, this was a question of professional ethics: ‘it wasn't ethical to become the intruder in that situation. So I stepped back and I refrained from saying anything’. This informant traverses roles between non‐person and team member as he attempts maintain social distance with his employer. Expressing emotional and moral ambivalence, PA06 described this as a struggle between ‘being genuine as a person’ and ‘being professional in what you're doing’.

## PAs protecting the private realm

A primary concern for some PAs was preserving their private realm. Whilst many disabled informants said that informal personal relationships were inevitable, PAs described efforts typical of non‐persons to limit reciprocal access to their personal information and backstage lives.

When speaking about her employer, informant PA07 said ‘her life is my life, I don't want my life to be hers’. Asked to explain this dynamic, PA07 described a relationship under strain:

She is not sharing a part of me, she is sharing herself. I don't want to share myself with her, I don't want to share myself with anybody if I don't have to, when you are disabled you have to.

Informant PA17 expressed similar concerns, and described her efforts to control information about her private life:

I don't say much … you have polite banter, but I think you still have to be careful what you say. They talk about your family. I talk about my family in general.

Explaining why she preferred to discuss her family in a superficial or ‘general’ way, PA17 said that disclosing personal information involved risks – ‘because you don't know what they will say to someone else’ – and that by restricting this information she was ‘protecting my own family’.

Informant PA23 recalled a situation where an employer became overly involved in her personal life: ‘she wanted to lead my life, live my family life with my problems, and to have control over it’. This informant responded by exerting tighter control over her personal information and through actively disclosing misinformation:

I would lie to her … it was little white lies, it doesn't hurt anybody, but I didn't tell her everything. Gradually I also started to tell her less about my life, it was more my life. [At] the beginning I thought we could be friends and share many things. No we can't, you have to keep this line.

Informant PA18 described a similar asymmetry of disclosure, saying ‘they want you to be part of their family, because you know so much about them, they want to know more about you’. This informant was clear about the need to protect her private life – ‘I'm sorry but this is my work situation and this is my home situation’ – and explained the risks at stake:

I know people who have got involved in family life, and if things do go wrong … they know my address, they know my name, and geographically we're very close … I don't want them to get the feeling that they can just pop around.

Informant PA11 described the acrimonious breakdown of a PA relationship, and in doing so, illustrated the consequences of full disclosure and admission to back stage regions. She described how the mother of the child she had supported continued to pursue grievances against her:

Afterwards I would get messages about how I'd let the family down, how awful I was to them … [They] were in loads of the same charity circles, all of the events we went to they were always there. It got to the point when we were all at an event and I was like “mum I have to leave, she won't stop.” She just wouldn't accept it and kept saying things.

In this example, the informant has ceded control over personal information and access to backstage regions of her life. There is little distinction between professional and personal domains. With the breakdown of the PA relationship, enmity is not limited to the relationships and spaces of front regions. Instead, conflict pollutes the private realm, and there is no longer any physical or emotional sanctuary remaining.

The PAs presented above assume roles akin to non‐persons. This discrepant role involves access to the personal information and backstage lives of their employer, whilst denying corresponding access. These are acts of self‐defence, which delimit professional realms and protect private lives.

## Discussion

Personal assistance relationships, defined narrowly, are the relationships that occur when disabled people employ another person directly. However, this definition falls far short of capturing the interpersonal dynamics of this ‘hybrid form of work and care’ (Ungerson 1999: 583). Personal assistance involves inherent tensions and ambiguities: part personal, part professional; instrumental, yet at the same time emotional. Previous studies have recognised these tensions, whether it be ‘paid friends’ (Woodin 2006) or ‘professional friendship’ (Christensen 2012), PA subverts normative role boundaries, as distinctions between public and private feelings, and between professional and personal actions do not obtain in any typical sense. The dramaturgical lens helps us to move beyond ambiguity and provides a clearer analysis of the micropolitics of interpersonal cooperation.

Personal assistance, as with other forms of intimate work (Milligan and Wiles 2010, Twigg *et al*. 2011), demands that disabled people invite PAs into the backstage regions of their life. The home space becomes another person's workplace, everyday tasks involve bodily intimacy, and typical working arrangements mean that both parties spend prolonged periods of time in one another's company. Admissions and disclosures of this kind mean that relationships develop, as PAs assume the roles of collaborators in the shared endeavour of their employer's independence. Informality often blossoms, out of preference but also necessity. Maintaining face is exhausting and disabled informants often do not want to, or cannot, continually maintain the face of an employer whilst in the backstage regions of their life.

Many informants, both employers and workers, said that informality led to valued personal relationships marked by mutual affection. These relationships resemble Goffman's team members in that both parties share personal information and access to backstage regions, whilst PAs in these roles hold a personal and emotional stake in the success of their collective endeavour – they care that their employer flourishes.

However, these relationships often involve risks. Some disabled informants reported that informal relationships were more likely to involve emotional entanglements, whilst many said that PAs were more likely to lower their standards in relaxed roles. For PAs in similar roles, the predominant risk is of giving too much of oneself to work, whether physically or emotionally. These findings suggest that despite the distinctiveness of PA, it has commonalities with other caring roles in that the ‘paradox of care’ prevails (Eustis and Fischer 1991). Informality is a prerequisite of good support, whilst also being a problematic aspect of support.

PA relationships are often deeply personal, but the extent to which employers and workers share in one another's private realms is often imbalanced. Such relationships follow Goffman's discrepant roles, and in particular, the ‘non‐persons’ of servants and service specialists. Ahlström and Wadensten have observed PA relationships marked by ‘incomplete mutuality’ whereby ‘the assistant includes the disabled person in the relationship but the disabled person does not include the assistant’ (Ahlström and Wadensten 2010: 185). In our study, we found contrary evidence as PAs control access to their personal spaces and limit the disclosure of personal information. Whilst some PAs spoke of deep emotional connections with their employers, many also described relationships characterised by asymmetrical disclosure: being privy to their employer's personal information, spaces and intimate activities, whilst simultaneously controlling access to their own. For most this represented an aspect of unseen and unacknowledged labour, which required skill and hard work.

Talk of blurred boundaries is common in PA literature (Glendinning *et al*. 2000). More clearly institutionalised relationships, such as friendship or colleagues, proceed with the individuals involved sharing broadly equivalent expectations of one another. Personal assistance, when shaped by asymmetrical disclosure, involves discord from the outset. Some employers and workers find this confusing and need time to understand the appropriateness of feelings, actions and settings within given relationships. Others will experience anxiety as the tacit boundaries that normally distinguish personal lives come to feel ill‐defined and open to transgression. Left unattended, the latter situation becomes unsustainable and will likely lead to conflict. For this reason, we agree with commentators who argue that PAs receive insufficient guidance about boundaries (Christensen 2012).

PAs and disabled employers need support to understand how far they wish to share in one another's lives, and to develop strategies that enable asymmetrical disclosure to work in ways that are acceptable and rewarding to both parties. The dramaturgical reading of PA we present lays the groundwork for this. Models of reflective practice commonly directed towards the health and social care workforce (Atkins and Murphy 1995) need now to be adapted and extended to PA. Disabled employers and their workers, particularly those new to PA, would benefit from understanding their relationship preferences, and the potential risks and rewards involved in these choices. This, we suggest, is vital, so that more disabled people may experience PA as a radically empowering, transformational and sustainable model of support.

## Conclusion

Personal assistance is a unique social relationship, which subverts typical interpersonal boundaries. Disabled employers and PAs often hold divergent views and preferences concerning the status of their relationships. Disabled employers and PAs need support to reflect upon the kinds of relationship they desire, and the implications of these preferences. This simple step would likely lead to more disabled people experiencing this transformational model of support in an empowering and sustainable way.
